# Glenoid Fracture Fixation Using an Acu-Loc Distal Radius Plate

**DOI:** 10.7759/cureus.60751

**Published:** 2024-05-21

**Authors:** Adam T Stammer, Prateek Saxena, Andrew P Dekker, Neil Ashwood

**Affiliations:** 1 Orthopaedic Surgery, Trauma & Orthopaedics, University Hospitals Derby and Burton, Derby, GBR; 2 Trauma and Orthopaedic Surgery, Trauma & Orthopaedics, University Hospitals Derby and Burton, Derby, GBR; 3 Trauma & Orthopaedics, University Hospitals Derby and Burton, Derby, GBR

**Keywords:** long-term follow-up after orthopedic surgery, distal end radius plating, open glenoid augmentation, glenoid reconstruction, scapula and shoulder trauma

## Abstract

Displaced fractures of the glenoid require surgical fixation. This poses multiple problems, including a difficult approach and achieving adequate reduction with current implants. We provide a surgical technical tip for fixing scapula neck and glenoid rim fractures with an Acu-Loc distal radius plate (Acumed, Weyhill, UK), illustrated with two recent case reports. Here, we present two cases of a 58-year-old female and a 51-year-old male presenting to a hospital following a fall, both sustaining an isolated right glenoid intra-articular fracture evident on plain radiographs. CT scans revealed a displaced and fragmented glenoid surface. A reverse Judet posterior approach facilitated exposure to enable the reduction of the glenoid, an uncommon approach. Current plate designs provide surgeons with limited options to fix complex fractures of the scapula and were not suitable here. The lateral scapula border and inferior glenoid have a similar anatomical shape to the distal radius. An Acu-Loc locking distal radius plate with a radial styloid plate was trialled and provided a good reduction to the fragmented glenoid. A distal radius plate can be a useful option to consider in complex scapula neck and glenoid rim fractures. A better understanding of glenoid shape will facilitate the further development of orthopaedic implants. Familiarity with various surgical approaches is needed to operate on these complex fractures.

## Introduction

Scapula fractures occur in only 3-5% of all upper limb fractures, and glenoid fractures constitute only 10-20% of all scapular fractures [1]. Most scapular fractures are treated non-operatively, but displaced glenoid fractures need to be surgically fixed for favourable outcomes [2]. Surgical fixation of these fractures can pose unique challenges owing to the difficult surgical approach, achieving anatomical reduction, intraoperative imaging, and an adequate implant. A range of implants is available in the market for fixing these complex fractures, which include locking compression plates, reconstruction plates, Y or T plates, calcaneus deformed plates, and microplates. Aculoc distal radius plates are commonly employed for the fixation of distal radius fractures and have favourable outcomes. The aim of study is to provide a surgical technical tip for fixing scapular neck and glenoid rim fractures using a distal radius plate.

## Case presentation

Case 1

**Figure 1:**
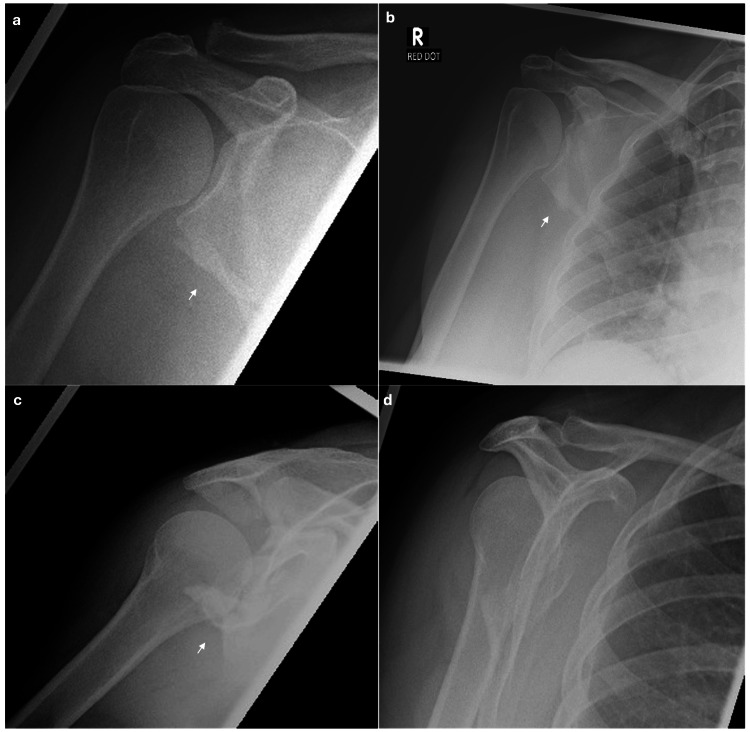
Pre-operative radiographs. a. Anterior-posterior shoulder X-ray. b. Anterior-posterior shoulder X-ray. c. Axial shoulder radiograph. d. Lateral shoulder X-ray. Figures 1a-1d show evidence of a comminuted intra-articular fracture of the glenoid with inferior displacement as indicated by the white arrow.

A 58-year-old female presented to our hospital due to a fall while walking through a set of patio doors. She fell with hand right arm outstretched to break the impact of the fall, resulting in immediate right shoulder pain. Prior to the fall, she was independently carrying out her activities of daily living. She did not have any significant medical comorbidities. Upon her presentation to the emergency department, her primary survey was clear. She sustained an isolated right shoulder injury as identified on a secondary survey. On initial examination, she was found to have significant bruising on the medial aspect of her upper arm. On palpation, she was tender over the scapular body and neck. All the movements of the right arm were painful. There was no neurological or vascular compromise on initial examination. Plain radiographs and CT of the right shoulder concluded a displaced comminuted intra-articular fracture of the right glenoid body with inferior displacement of the fractured glenoid fragment (see Figure 1). Vital signs were recorded within normal range, and laboratory results were unremarkable for her age.

She had a broad arm sling for analgesia and comfort in the emergency department. Following the CT scan, she underwent open reduction and internal fixation of the right glenoid fracture a week after her injury.

Surgical Technique

After administration of general anaesthesia and prophylactic antibiotics, she was positioned in a lateral decubitus position with all pressure points padded. The right arm was prepped, and the arm abducted freely on the armrest so that it could be easily manipulated during the surgery.

**Figure 2:**
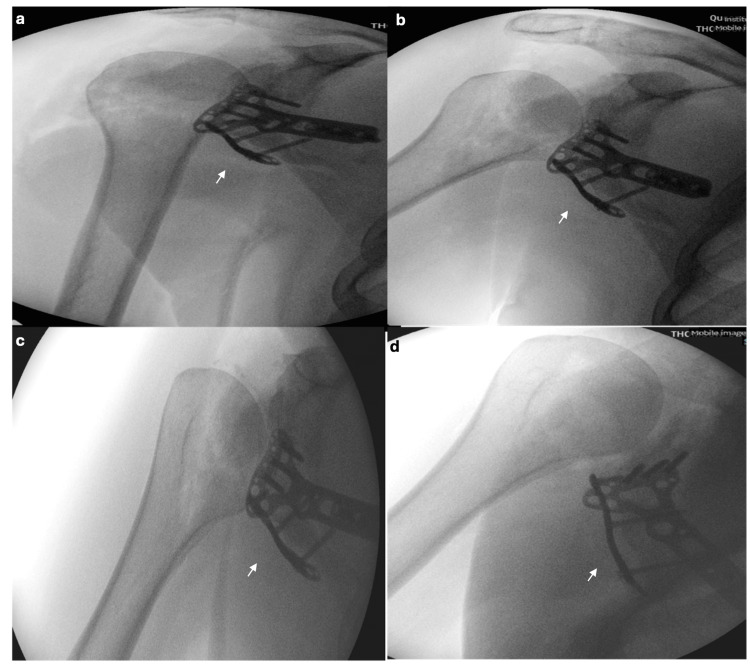
Intra-operative X-rays taken with an image intensifier machine. a. Anterior-posterior X-ray with the arm adducted. b. Anterior-posterior X-ray with the arm adducted. c. Lateral X-ray with the arm in adduction. d. Lateral X-ray with the arm in abduction. Figures 2a-2d show that the glenoid fracture has been successfully reduced and internally fixated with the use of a distal radius plate and screws. The fixation remained stable on anterior-posterior and lateral views with the abduction-adduction movement of the arm (as indicated by arrows).

A reverse Judet posterior approach to the scapula was used, and an L-shaped incision was made towards the shoulder tip curved towards the axilla. Full-thickness skin and the subcutaneous flap were reflected, medially exposing the posterior third of the deltoid, infraspinatus, and teres minor muscles. The deltoid was split in line towards its insertion, released from the scapula spine, and reflected laterally and distally on the fascial cuff. The infraspinatus and teres minor were tagged with stay sutures with the posterior capsule and tenotomised, followed by an insertion into the greater tuberosity. These were then reflected onto the scapula; the axillary and suprascapular nerves were protected throughout. Intraoperatively, we noted a comminuted intra-articular glenoid fracture extending into the neck, which was completely dissociated from the scapula body with a further scapular body fracture. Fracture hematoma was cleared, and the fracture fragments were reduced anatomically and held with K-wires. We attempted to compress the glenoid fracture with an Acutrak screw (Acumed, Weyhill, UK), but the screw cut out from the distal bone. The current scapular plates available were unsuitable to fix the fracture owing to its configuration. We attempted to stabilise the posterior glenoid fracture with a broad Acu-Loc distal radius plate (Acumed, Weyhill, UK) and achieved very good hold and stability. The distal radius plate was placed in buttress mode onto the glenoid fragment and fixed to the blade of the scapula. To augment this fixation, the radial styloid plate from the same wrist plating system was used and interlocked with anterior-posterior screws from the distal radius plate. The blade of the scapula had significant comminution, and this defect was filled with synthetic bone graft chips (Vitoss, Stryker, UK) and AttraX putty (NuVasive, Elstree, UK). The superior glenoid fragment and posterior labrum were augmented onto the plate using a 1.45 mm JuggerKnot anchor (Zimmer Biomet, UK). Satisfactory reduction of fracture and position of plates was confirmed using an image intensifier in the theatre (as shown in Figure 2). The wound was washed with copious amounts of normal saline. The teres minor and infraspinatus were repaired back to a lesser tuberosity tissue cuff with the FibreWire suture (Arthrex, Sheffield, UK) and 1.45 mm JuggerKnot anchor. The deltoid was repaired back to the fascia using FibreWire. The skin was closed with monocryl, dressed with mepilex, and held in an external rotation sling.

**Figure 3:**
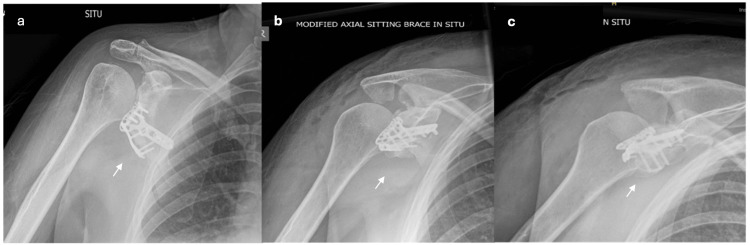
Day one post-operative radiograph of the right shoulder. a. Anterior-posterior view, b. axial view, and c. modified axial view. Figures 3a-3c display the maintained reduction and position of the distal radius plate at post-operative day one (as indicated by arrows).

**Figure 4:**
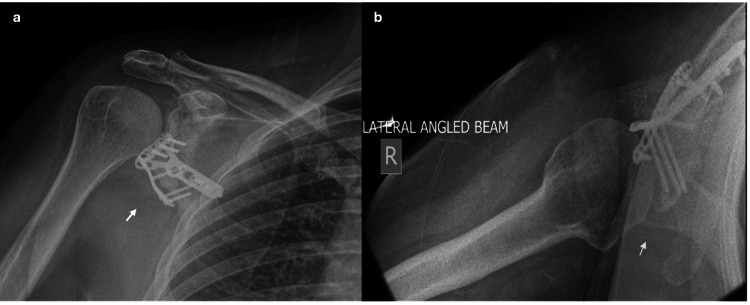
Six-week post-operative radiographs of the right shoulder. a. Anterior-posterior right shoulder X-ray. b. Axial right shoulder radiograph. Figures 4a-4b show a well-maintained reduction and stable position of the distal radius plate six weeks post-operatively (as indicated by arrows).

**Figure 5:**
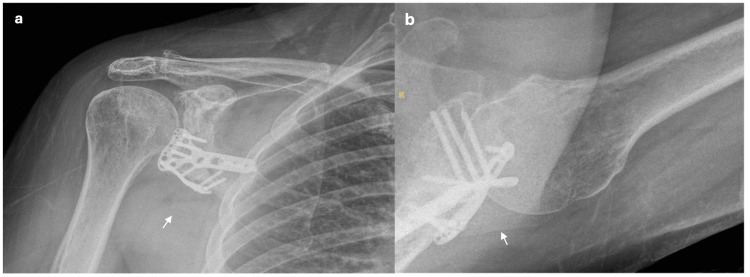
Seven-month post-operative shoulder radiographs. a. Anterior-posterior right shoulder X-ray. b. Axial right shoulder radiograph. Figures 5a-5b show a well-healed fracture with the maintained position of the distal radius plate seven months post-operatively (as indicated by arrows).

In the post-operative period, the patient was advised to keep the arm non-weight bearing with gentle passive range of motion exercises in a sling within the safe zone (neutral to 45 degrees of forward elevation). In the first four weeks, internal and external rotation of the arm was avoided using a shoulder sling to protect the tenotomy repair. This was followed by gentle pendulum exercises and passive physiotherapy for the next two weeks. Radiographs were taken one day, two weeks, and six weeks post-operatively, which showed the position of the distal radius plate remained stable, and the fracture reduction was maintained, as shown in Figures 3-4. At six weeks, she was allowed to be out of the sling, and active mobilisation of the arm with physiotherapy was commenced. After seven months she was pain-free, there was some residual stiffness in the shoulder, and she had 160° of active flexion, 60° of abduction, and 30° external rotation present. Final radiographs at seven months showed a well-healed fracture (Figure 5).

Case 2

**Figure 6:**
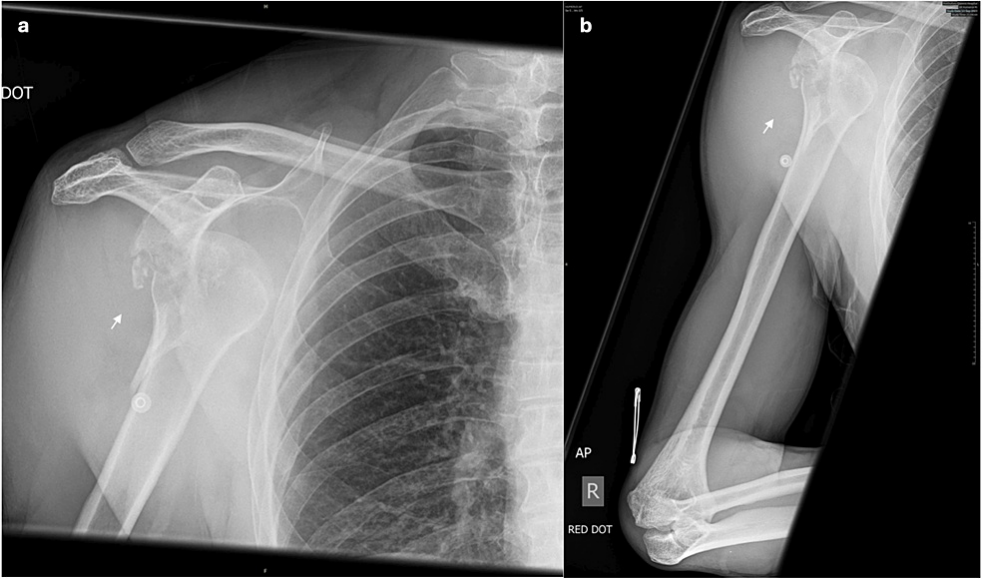
Pre-operative radiographs. a. Anterior-posterior X-ray. b. Humerus X-ray. Figures 6a-6b show a fracture dislocation of the glenohumeral joint with evidence of a comminution and dorsal displacement of fracture fragments (white arrows).

**Figure 7:**
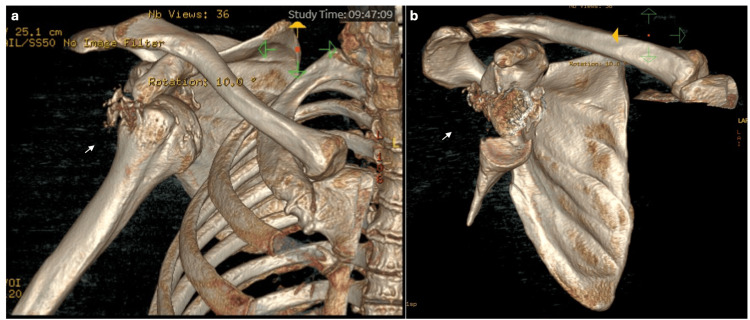
3D reconstruction of right CT shoulder imaging. a. 3D reconstruction showing the anterior view of the glenohumeral joint. Imaging shows a complex comminuted fracture of the glenohumeral joint and posterosuperior fracture of the humeral head. b. 3D reconstruction from CT shoulder, humeral head removed to better view the glenoid. The image displays evidence of a complex comminuted fracture at the anteroinferior aspect of the glenoid with involvement of the intra-articular surface (as shown by white arrows).

A 51-year-old male was brought to the emergency department by ambulance following a collapse. The patient had little memory of the events. The fall was witnessed by bystanders who reported the patient appearing vacant, followed by a collapse and subsequent tonic-clonic seizure activity. The patient fell onto the right shoulder during this collapse. He had a known history of alcohol and substance misuse and had previously presented with associated withdrawal seizures. Prior to this incident, he admitted to using cocaine in the morning and denied any alcohol intake. He had no other comorbidities and took no regular medications. He was previously independent for all activities of daily living. Primary and secondary surveys found that there was an isolated right shoulder injury. On examination, the shoulder appeared to be anteriorly dislocated. Movement was limited by pain. No neurovascular compromise was observed. The pain was managed in the emergency department with a broad arm sling and analgesia. Shoulder X-rays showed a fracture dislocation of the glenohumeral joint with evidence of a comminuted fracture of the scapula blade and dorsal displacement of fragments (Figure 6). No abnormalities were found on the CT head. A CT was arranged to better evaluate this glenoid fracture. The CT showed a complex comminuted fracture at the anteroinferior aspect of the glenoid with a large, displaced fragment at the lateral angle of the scapula and involvement of the glenoid intra-articular surface. The humeral head had a severe posterosuperior fracture extending to the greater tuberosity, as shown in Figure 7. Following these results, it was decided to plan for a complex reconstruction and delay any immediate manipulation of the shoulder joint under anaesthesia.

Surgical Technique

**Figure 8:**
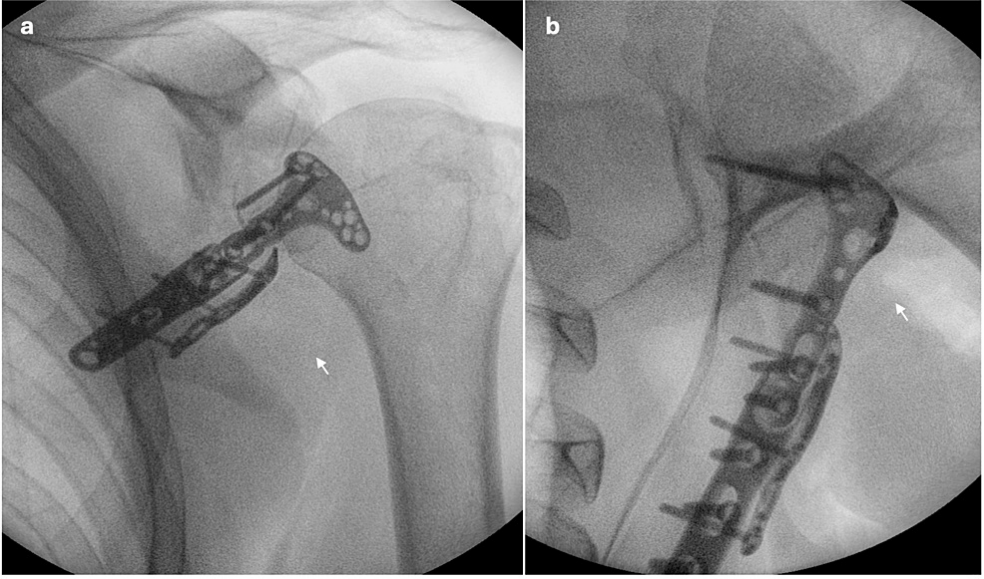
Intra-operative X-rays taken with an image intensifier machine. a. Posterior radiograph. b. Axial radiograph. Figures 8a-8b show fixation with a distal radius plate and screws provided good reduction of the fracture (white arrows).

The patient underwent general anaesthesia and interscalene nerve block. Antibiotic prophylaxis was given (cefuroxime 1,500 mg intra-operatively, followed by two further doses of cefuroxime 750 mg at eight-hour intervals). The patient was placed in a lateral decubitus position, and pressure points were protected. The right arm was prepared with alcoholic iodine, and the surrounding area was covered with surgical drapes. A combined deltopectoral and reverse Judet posterior approach was used to assess the fracture. The infraspinatus and teres minor planes were developed. The axillary and suprascapular nerves were then identified and protected. The infraspinatus and teres minor were tenotomised at the lesser tuberosity, with the shoulder capsule on stay sutures. Exposure of the inferior glenoid identified a comminuted intra-articular fracture of the glenoid with glenoid neck fracture and complete dissociation from the scapula border. A further scapula border fracture was also seen. The fractures were cleaned and reduced initially with Kirschner wires. The best hold was achieved with an Acu-Loc distal radius plate, which was fixed to the posterior glenoid and the blade of the scapula. This was augmented with a radial styloid plate to interlock the anterio-posterior screws from the wrist plate. Fracture reduction and metalwork position were confirmed to be satisfactory using a mobile image intensifier, as shown in Figure 8. The wound was washed in normal saline. The teres minor and infraspinatus were repaired back to a lesser tuberosity tissue cuff with the FibreWire suture and 1.45 mm JuggerKnot anchor. The deltoid was repaired back to the fascia using FibreWire. The greater tuberosity fracture appeared stable and was therefore not repaired at this time. The skin was closed with monocryl, dressed with mepilex, and held in an external rotation sling.

Following the operation, intraoperative images were reviewed, and there was concern over the lateral most screw extending too far into the glenoid joint. This was confirmed on CT, and the patient underwent a further procedure five days later to remove this screw. The greater tuberosity was exposed, haematoma evacuated, and repaired using the transosseous No. 2 fibrewire suture. Mobile intensifier images were satisfactory.

**Figure 9:**
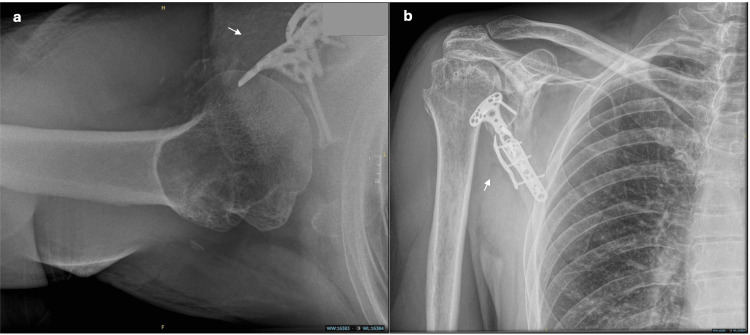
Three-week post-operative radiographs. a. Axial shoulder X-ray. b. Anterior-posterior shoulder X-ray. Figures 9a-9b show the distal radius plate remained in a stable position, and fracture reduction was well-maintained three weeks post-operatively (white arrows).

**Figure 10:**
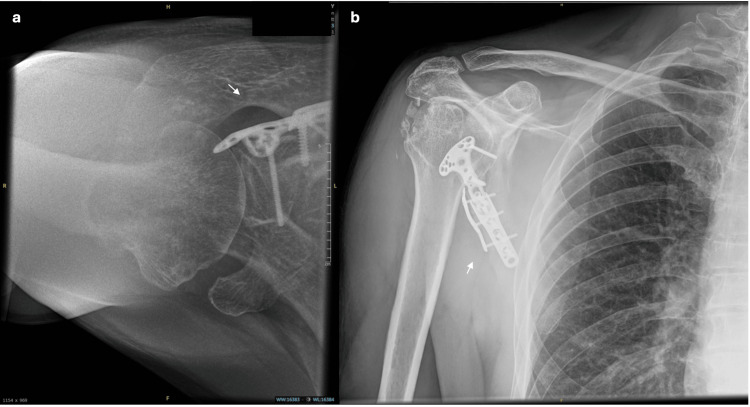
Three-month post-operative radiograph. a. Axial shoulder X-ray. b. Anterior-posterior shoulder X-ray. Figures 10a-10b show evidence of a good fracture union three months post-operatively (white arrows).

Post-operatively, the patient was advised for a gentle, passive range of motion in a sling, with movement allowed from a neutral position to a maximum of 45 degrees forward elevation. Internal and external rotation movements were avoided for the first four weeks to protect the tendon repair. Pendular exercises and passive physio were commenced at four weeks, and active mobilisation without a sling was allowed at six weeks. The patient was reviewed in the clinic three weeks and three months post-operatively. On review, X-rays were satisfactory, showing a good union of fractures, as shown in Figures 9-10. He remained neurovascularly intact with no pain. He had some residual stiffness in abduction and extension, which is expected to improve with further physiotherapy and strengthening. There was mild pain over the anterior and posterior shoulder. A further follow-up has been arranged at six months post-operatively.

## Discussion

Anatomy of the scapula and glenohumeral joint

The glenohumeral joint is formed by the articulation of the humeral head with the glenoid fossa of the scapula. The glenoid cavity is a relatively shallow depression in the lateral scapula neck, which can be shaped in a pear or oval configuration [3]. The head of the humerus is large in relation to the glenoid cavity. This discrepancy allows for the formation of a synovial joint with a wide range of movement, a feature that is important for the function of the upper limb. This additional mobility, however, comes at the expense of joint stability [4]. The glenohumeral joint compensates for this inherent instability with a combination of static and dynamic mechanisms. Firstly, the glenoid fossa is surrounded by a rim of fibrocollagenous tissue, the glenoid labrum. This tissue functions to effectively deepen the cavity and provide passive stabilisation. The glenohumeral joint is surrounded by a fibrous sheath, the glenohumeral capsule, which is attached laterally at the anatomical neck of the humerus and medially to the edge of the glenoid fossa. The capsule is thickened in three regions, forming the superior, middle, and inferior glenohumeral ligaments, which act to further improve joint stability [4]. The muscles of the rotator cuff provide further balance to the joint by producing opposing forces; for example, the counter-actions of the subscapularis and infraspinatus muscle pull up the humeral head anteriorly and posteriorly, respectively, and create tension to draw articulating structures into closer proximity [4]. Despite these mechanisms, the glenohumeral joint remains prone to injury through trauma, resulting in joint dislocations and fractures.

Epidemiology

Scapula fractures account for approximately 1% of all fractures and 3-5% of those around the shoulder [5]. The incidence of scapula fractures appears to be increasing. A recent review of the US National Trauma Data Bank showed that scapula fracture rates doubled from 1% to 2.2% of all fractures [6]. The scapula can be fractured in multiple regions. A review by Tuček et al. found that the most common location of scapula fractures was within the body, occurring in 52% of cases [1]. This was followed by glenoid fractures, identified in 21% of cases, while the least common region was the scapula neck in 8% of cases [1]. Scapula fractures are typically associated with blunt-force, high-energy trauma. Fractures to the scapula body are more associated with direct trauma, while injuries to the glenoid and scapula neck are most often a result of indirect trauma where the humeral head is impacted into the glenoid fossa or secondary to trauma from a shoulder dislocation [7].

Patients can be divided into two main groups; those who fracture the scapula as a result of high-energy trauma tend to be male and younger (< 60 years old), while those who suffer low-energy trauma tend to be older and female [2]. Due to the nature of the injury, the first group often has additional injuries, including upper limb, thoracic, and pelvic injuries, while the second group is more prone to fractures of the proximal humerus [2]. There is evidence to suggest that, in a group of patients sustaining high-energy trauma, those with a scapula fracture may in fact have a lower mortality than those without [8]. This is likely due to the scapula absorbing some of the force resulting in a fracture, thereby protecting from other injuries to the thorax such as pneumothorax, which can increase mortality. Finally, a growing group of fractures are those involving the spine of the scapula. These fractures are associated with reverse shoulder arthroplasty and most commonly occur at the base of the acromion [2].

Presentation and assessment

Patients with scapula fractures will typically present with localised pain around the shoulder with the arm supported and held in an adducted position. Examination findings are often limited to swelling, bruising, and localised pain on palpation around the shoulder. Range of movement is limited by pain, and abduction is most restricted [9]. It is crucial to appreciate that scapula fractures are a marker for severe injury, with more than half of patients having additional injuries and many having experienced high-energy trauma [10]. It is therefore important to conduct a full primary and secondary survey in these patients with a high index of suspicion for associated injuries. A careful examination is also needed to review for injuries to the nearby brachial plexus and vascular structures [9].

Many patients presenting to the emergency department will receive an AP chest X-ray, which can be the first opportunity to diagnose a fracture [2]. This investigation has a low sensitivity for identifying scapula fractures with one study showing that up to 43% of fractures may be missed for multiple reasons including poor visualisation of the scapula due to overlying tissues, non-inclusion in the study, and failure to identify the fracture by the observer [11]. A dedicated shoulder trauma series, including a lateral scapula (Y), Grashey, and axial view, allows for better diagnosis of the scapula, clavicle, and glenohumeral joint injuries [10]. As scapula fractures are commonly associated with high-energy trauma, many patients have a series of CT trauma scans. This investigation has good sensitivity for identifying scapula fractures and has therefore improved the detection rate [2,3]. A dedicated shoulder CT can be a beneficial subsequent investigation in complex fractures as the thinner slices allow for better fracture visualisation and 3D reconstruction, which can be useful in pre-operative planning [2,9].

Classification of scapula fractures

There are multiple systems to classify scapula fractures. The first and simplest was from Jean-Louis Petit who divided fractures affecting the body, neck, and processes of the scapula [2]. More recently, there is the Ideberg classification [12], which is the most used classification in research. Type I is a fracture of the glenoid rim, with type 1a affecting the anterior region and type Ib affecting the posterior glenoid rim. Type Ia was found to be the most common scapula fracture by Ideberg and is commonly associated with a shoulder dislocation (bony Bankart). Types II and III are fractures of the glenoid, which exit inferiorly and superiorly, respectively. Type IV is a glenoid fracture that exits medially, affecting the body of the scapula. Type V is a combination of II and IV, while type 6 represents severe comminution [12,13]. The AO Foundation and Orthopaedic Trauma Association (AO/OTA) system is also widely used and classifies fractures into those affecting the body, the processes, and intra-articular fractures of the glenoid fossa. This system has the added benefit of better describing glenoid fractures that can be beneficial in helping inform management decisions [3,14]. There are ongoing discussions in the literature regarding the best way to classify these fractures, and further systems are therefore likely to be developed as the consensus around how to manage these heterogeneous fractures evolves over time.

Indications for surgery vs non-operative management

The majority of scapula fractures (> 80%) are managed conservatively with good functional outcomes [15]. Isolated fractures to the scapula body, neck, and non-displaced fractures to the acromion, coracoid process, and scapula spine are typically managed conservatively [3,15]. Conservative management involves pain relief, immobilisation, and support with a sling for two weeks [15]. This is followed by physiotherapy increasing from passive to active range of motion exercises and then strengthening exercises starting after six weeks. Recent reviews have shown that conservative management provided excellent or good outcomes in most patients with the above fractures [15].

Indications for operative treatment remain controversial and are often poorly defined. There are inconsistent parameters in the existing literature, and many publications apply available criteria for surgery inconsistently, meaning that drawing conclusions is difficult [2].

Surgery is always required for open fractures, those with evidence of lateral column displacement, and disruption at two points of the scapula suspensory complex [2,5]. Indications can be further divided into intra-articular and extra-articular. Intra-articular markers are an intra-articular gap of > 3-5 mm and articular rim fractures that involve more than 25% and 33% of the anterior and posterior rims, respectively [16]. Extra-articular indications are medialisation of the glenoid fragment > 20 mm, a glenopolar angle <22° on a radiograph, and angular deformity >45° on a scapular Y radiograph [2,16,17]. It is important for the surgeon to consider these parameters in the context of patient factors, including age, hand dominance, activity level, and profession before considering surgical intervention [16]. A study by Jones et al. compared the outcomes of patients with operative and non-operative management. Although the group of patients receiving surgery had more severe injuries, it was found that outcomes for both cohorts were equally good [18]. Martika et al. compared conservative and surgical management and found that patients with surgical management had a better functional outcome, with minimal complications in comparison to the conservative group [19]. These studies suggest that surgery has a beneficial role in severe injuries, although direct comparisons are difficult to make; further research is therefore required to directly compare these treatment strategies and define indications for surgery more clearly.

Surgical approaches and implants

Multiple approaches are used to access fractures of the scapula depending on the location. Fractures of the anterior glenoid, most commonly Ideberg type Ia, are accessed with the patient in a beach chair position through the anterior shoulder via a deltopectoral approach if open reduction and internal fixation are necessary; otherwise, percutaneous or arthroscopic methods can be attempted [13]. Fractures to the body of the scapula or posterior scapula neck are commonly accessed via a Judet approach or modified Judet approach if only more limited access to the lateral column is needed [13]. As in cases 1 and 2 seen here, fractures of the glenoid articular surface can pose a particular problem as access is difficult with the typical Judet approach [20]. Here, we utilised a reverse Judet incision in both cases, which provided good exposure to the fracture site [20]. In case 2, an additional deltopectoral approach was also utilised to facilitate greater access. It is therefore very important for the surgeon to be familiar with these approaches when managing scapula fractures and choose the correct approach for the fracture.

**Figure 11:**
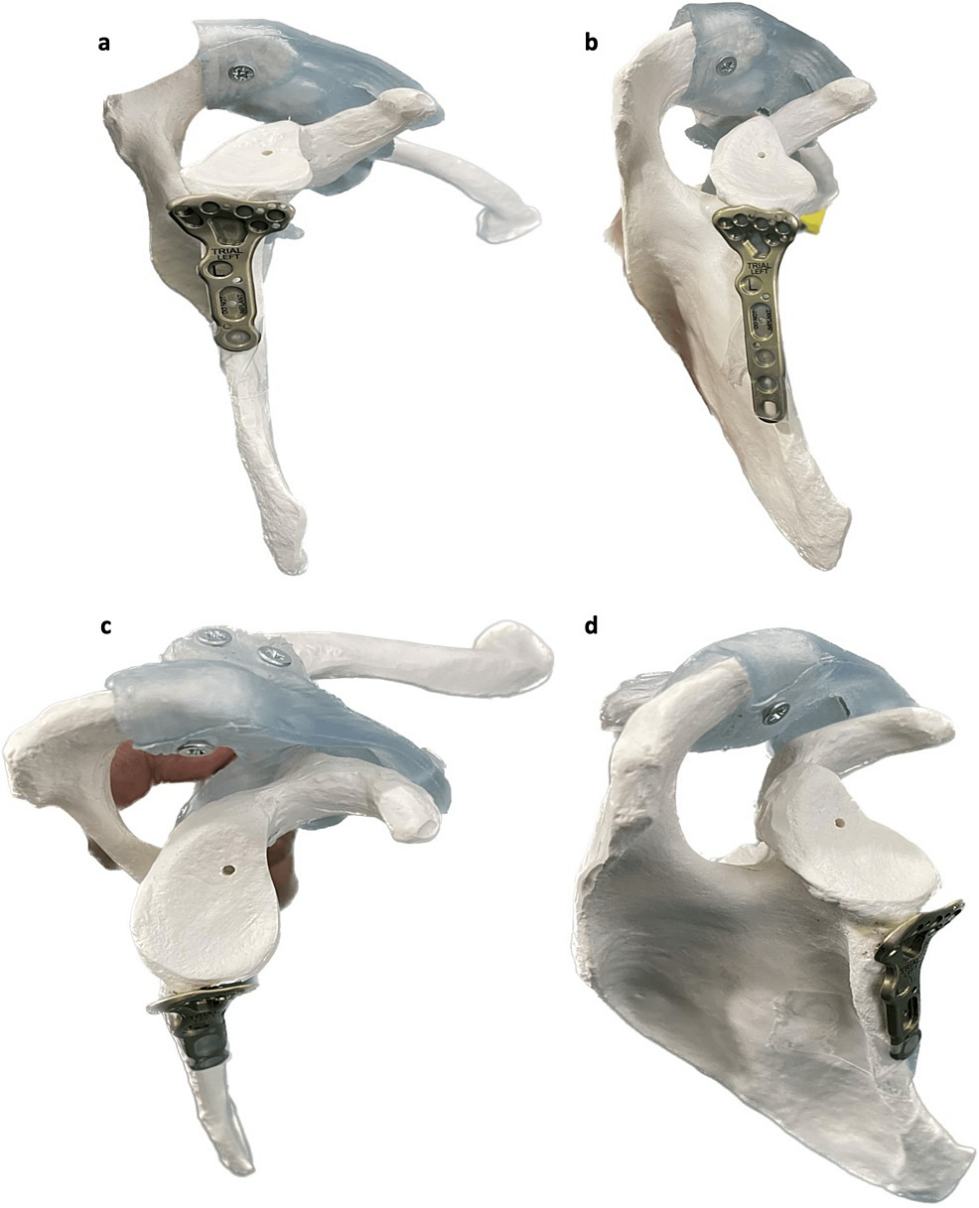
Distal radial plate positioned over the blade of the scapula on the anatomical model. a. Inferior lateral view. b. Lateral view. c. Superior lateral view. d. Inferior posterior view. Figures 11a-11d represent the location of the distal radius plate used for fixation of the displaced glenoid fractures in cases 1 and 2 reported here.

There are a number of implant options that can be utilised for scapula fracture fixation. These include compression screws, straight and Y-type locking plates, T plates, reconstruction plates, microplates, and more recently custom-designed plates. Despite this variety, there can be significant difficulties in achieving adequate, stable reduction of fractures, especially in intraarticular fractures of the glenoid. In both cases here, current implants were unsuitable due to the fracture configuration, and sufficient fixation was not achievable with the placement of a compression screw alone. In both of these cases, a distal radial plate provided excellent hold via the buttressing effect from the plate and allowed stable fixation of both fractures. Figure 11 shows an anatomical model of the scapula with a distal radius plate positioned to fix a glenoid fracture. In case 1, the patient made a good recovery with pain well controlled and a good range of movement available at seven months post-op. In case 2, the patient was assessed at three months, fractures were shown to be well-healed, and the patient had some residual stiffness, but is expected to improve with further physiotherapy and is awaiting further follow-up. These cases show that a distal radius plate can be successfully utilised to provide good fixation of displaced glenoid fractures; however, there is still a need for further implant design to accommodate the variety of fracture types seen in these injuries.

## Conclusions

Scapula fractures are an uncommon injury, accounting for approximately 1% of fractures, and are often associated with major trauma. Generally displaced fractures need to be fixed surgically for good outcomes. Further research is needed to better establish and define indications for surgery and allow better comparison between conservative and operative management. Here, we presented two cases where current implants were unsuitable for fixation and where a distal radius plate was successfully used to provide good fixation as an alternative. Results on follow-up appeared positive with case 1 making a good recovery and case 2 experiencing only some residual stiffness, which is expected to improve with further physiotherapy. Surgeon familiarity with various surgical approaches to the shoulder is important to allow good access, which is important in repairing these injuries. A distal radius plate is therefore a good alternative to be considered in similar scenarios. Further innovation is required to produce specialised implants that can be used in these cases.
